# Liquid biopsy provides new insights into gastric cancer

**DOI:** 10.18632/oncotarget.24540

**Published:** 2018-02-21

**Authors:** Camila Tavares Uchôa Guimarães, Nina Nayara Ferreira Martins, Kelly Cristina da Silva Oliveira, Caroline Martins Almeida, Thayanne Macedo Pinheiro, Carolina Oliveira Gigek, Sandro Roberto de Araújo Cavallero, Paulo Pimentel Assumpção, Marília Arruda Cardoso Smith, Rommel Rodríguez Burbano, Danielle Queiroz Calcagno

**Affiliations:** ^1^ Residência Multiprofissional em Oncologia, Hospital Universitário João de Barros Barreto, Universidade Federal do Pará, Belém, PA, Brazil; ^2^ Núcleo de Pesquisas em Oncologia, Universidade Federal do Pará, Belém, PA, Brazil; ^3^ Disciplina de Genética, Universidade Federal de São Paulo, São Paulo, SP, Brazil; ^4^ Disciplina de Gastroenterologia Cirurgica, Universidade Federal de São Paulo, São Paulo, SP, Brazil; ^5^ Laboratório de Biologia Molecular, Hospital Ophir Loyola, Belém, PA, Brazil

**Keywords:** circulating tumor cells, circulating tumor DNA, circulating microRNAs, circulating long non-coding RNAs, precision medicine

## Abstract

Liquid biopsies have great promise for precision medicine as they provide information about primary and metastatic tumors via a minimally invasive method. In gastric cancer patients, a large number of blood-based biomarkers have been reported for their potential role in clinical practice for screening, early diagnosis, prognostic evaluation, recurrence monitoring and therapeutic efficiency follow-up. This current review focuses on blood liquid biopsies' role and their clinical implications in gastric cancer patients, with an emphasis on circulating tumor cells (CTCs), circulating tumor DNA (ctDNA) and circulating non-coding RNAs (ncRNAs). We also provide a brief discussion of the potential and limitations of liquid biopsies use and their future use in the routine clinical care of gastric cancer.

## INTRODUCTION

Among the various types of cancer that affect humans, gastric cancer (GC) is the fifth most frequent tumor type and the third leading cause of cancer death worldwide [1]. The high mortality rate presents a major clinical challenge because most GC cases are diagnosed at an advanced stage with poor prognosis, limited treatment options, and frequent metastasis and recurrence [2].

The sensitivity and specificity of current blood biomarkers for GC are insufficient to define the diagnosis and prognosis. In general, a GC diagnosis relies on an upper digestive endoscopy, an invasive procedure with a relatively high cost and unusual but serious adverse events [3, 4]. In addition, a single tumor-biopsy may not represent the intratumoral heterogeneity and can contribute to treatment failure and drug resistance [5]. Thus, new promising analyses of liquid biopsies should be further explored and validated for use in a clinical setting in GC patients.

Liquid biopsies have emerged as a new strategy for use in the clinical treatment of different cancer types to provide early disease detection, determine the tumor genomic profile, monitor treatment responses, assess the emergence of therapy resistance, quantify minimal residual disease, and perform real-time cancer management [6].

Originally, the term liquid biopsy had been assigned only to the investigation of circulating tumor cells (CTCs) in the blood of patients with cancer, but has now been extended primarily to include the analysis of circulating tumor DNA (ctDNA) and circulating non-coding RNAs (ncRNAs) [7].

In the current review, we highlight the recent advances in liquid biopsies and examine how different forms of liquid biopsies can be exploited to improve GC patient care. We argue that they should ultimately be integrated into clinical practice for GC management, with a focus on CTCs, ctDNA and circulating ncRNAs.

## CIRCULATING TUMOR CELLS (CTCs)

Circulating tumor cells (CTCs) have been identified as predictive and prognostic biomarkers that are useful for clinical approaches. They can determine risk for metastasis and can provide real-time monitoring of the therapeutic response in cancer patients [7]. Table 1 summarizes known CTCs as potential prognostic biomarkers and their utility for the evaluation of therapeutic efficacy in GC [8–29].

**Table 1:** CTCs and their clinical implication in GC patients

Markers	Methodology	Samples	Country	Clinical Implications	References
CEA, CK19, hTERT and MUC1 mRNA	qRT-PCR	64 GC	China	The membrane array-based method is a potential tool for detecting CTCs for early diagnosis and postoperative surveillance.	[8]
EpCAM, CK8, CK18, CK19 and CD45-	CellSearch	41 GC	Japan	CTCs number associated with advanced stage, peritoneal dissemination, metastasis and poor survival.	[9]
BIRC5, CEA, CK19 and VEGF	qRT- PCR	70 GC	Italy	BIRC5 has a significant prognostic value to the current TNM staging system.	[10]
EpCAM, CK8, CK18, CK19 and CD45-	CellSearch	52 AGC	Japan	CTCs enumeration may be useful as a marker for determining response to S1-based or paclitaxel regimens in AGC.	[11]
piR-651 and piR-823	qRT-PCR	93 GC	China	Levels of piR-651 and piR-823 could be useful to diagnosis GC with high sensitivity and specificity.	[12]
EpCAM, CK8, CK18, CK19 and CD45-	CellSearch	265 GC	Japan	CTCs associated with significantly worse OS.	[13]
EpCAM, CK8, CK18, CK19, CD45- and HER2	CellSearch	34 GCM	Japan	HER2 status of CTCs might be helpful for stratification of HER2-directed therapy.	[14]
CD44+ and CD45-	FACS	31 GC	China	CD44+/CD45- CTCs were associated with stronger malignant behavior and relatively sensitive to fluorouracil, cisplatin and paclitaxel, but relatively resistant to irradiation, oxaliplatin, cetuximab and trastuzumab.	[15]
EpCAM, CK8, CK18, CK19, Vimentin, Twist and CD45-	CanPatrol (RNA-ISH)	44 GC	China	Mesenchymal CTCs have a potential relevance to therapy response and can be useful on a therapeutic resistance.	[16]
Chromosomes 7 and 8	FISH	8 AGC	China	Reduction in CTCs count showed beneficial results to the patients treated with docetaxel/oxaliplatin/5-FU (DOF) regimen plus bevacizumab.	[17]
EpCAM, MUC1, *KRT19*, *MUC1*, *CEACAM5, EPCAM* and *BIRC5*	Immunomagnetic and RT-PCR	62 AGC	Germany	A combination of immunomagnetic separation of CTC followed by a real-time RT-PCR analysis of *KRT19*, *MUC1*, *EPCAM*, *CEACAM5* and *BIRC5* can serve as a prognostic tool for PFS and OS in patients with AGC.	[18]
CD133 and ABCG2	Flow cytometry and Immunomagnetic	36 GC	China	Presence of CD133 in bloodstream is potentially correlated with potentially be used as a marker of CTCs.	[19]
EpCAM, CK8, CK18, CK19, CD45-, CD19, CD20, CD40, CD44, CD133, CEA and HLA	CellSearch and Flow cytometry	42 AGC	Japan	CD44 is an appropriate biomarker of tumorigenic cells on peripheral blood.	[20]
EpCAM, CK8, CK18, CK19 and CD45-	CellSearch	136 AGC	Japan	Detection of CTCs was an independent predictor of a shorter PFS and could be a useful biomarker in the selection of patients who require intensive treatment in AGC. In addition, combined status of CTC and CY would be useful in selecting patients for radical gastrectomy.	[21]
OBP-401	FP-CTC Assay	37 GC	Japan	The number of CTCs (S-GFP+ cells) was relatively high in samples from GC patients who had received postoperative chemotherapy. However, no significant association between the change in the number of CTCs, treatment or prognosis in gastric cancer patients who underwent curative surgery.	[22]
CK4, CK5, CK6, CK8, CK10, CK13, CK18, CD45- and Chromosome 8	SE-iFISH	31 AGC	China	Aneuploidy of chromosome 8 in CTCs is associated with a poor prognosis.	[23]
EpCAM, CK7, CK18, CK19,CK20, CD45-, CD68, MUC1, HER2 and EGFR	MetaCell	22 GC	Poland	Higher sensitivity of CTC detection could be using a cytomorphological and molecular analysis.	[24]
EpCAM, CK8, CK18, CK19 and CD45-	CellSearch	136 GC	China	Post-treatment CTCs levels can help to evaluate therapeutic response and predict their prognosis in patients with AGC.	[25]
EpCAM, CK8, CK18, CK19, CD45- and c-MET	CellSearch and Immunomagnetic	7 GEA	USA	c-MET CTCs might be useful as a predictive biomarker for c-MET directed therapies.	[26]
EpCAM, CK and CD45-	FAST-disc	116 GC	Korea	Potential role of FAST-based CTC detection as an early diagnostic biomarker of GC.	[27]
Vimentin, CK8, CK18, CK19, CD45- and CA125	ISET	86 GC	China	CTCs could be divided into epithelial CTCs, epithelial/mesenchymal CTCs, and mesenchymal CTCs, whereas CTM could be divided into two subpopulations, including mesenchymal CTM and partially mesenchymal (epithelial/mesenchymal) CTM. Moreover, CTM were a independent predictor of worse PFS and OS in stage IV patients.	[28]
EpCAM, CK8, CK18, CK19, CD45- and HER2	CellSearch and IF-FISH	118 GC	Japan	IF-FISH method is applicable for select patients for trastuzumab therapies.	[29]

AGC: advanced gastric cancer; CTM: circulating tumor microemboli; CY: peritoneal lavage cytology; EMT: epithelial-mesenchymal transition; FACS: fluorescence activated cell sorter; FAST: fluid-assisted separation technique; GC: gastric cancer; GCM: gastric cancer with metastasis; GEA: gastro-esophageal adenocarcinoma; IF-FISH: immunofluorescence integrated with immunostaining-fluorescence in situ hybridization; ISET: isolation by size of epithelial tumor cells; PFS: progression-free survival; OS: overall survival; SE-iFISH: enrichment (SE) integrated with immunostaining-fluorescence *in situ* hybridization; RNA-ISH: RNA *in situ* hybridization.

In general, CTCs are rare in peripheral blood circulation and are found at a concentration of less than 5 CTCs per 7.5 ml of blood [30]. Furthermore, these CTCs originate from either primary or metastatic tumors [31], present a heterogeneous population and express the antigenic or genetic characteristics of a specific tumor type [32].

Early studies of CTCs characterized them as nucleated cells that express markers of epithelial cells EpCAM and cytokeratin 8, 18, and 19 (CK8, CK18, CK19), but are negative for CD45 (CD45-) [33]. Recent studies have described subpopulations of CTCs undergoing the epithelial mesenchymal transition (EMT) that may show decreased expression of EpCAM and cytokeratin with potential overexpression of mesenchymal markers, including Vimentin and Twist [34, 16]. In addition, it is also possible for these CTCs to undergo the reverse process, termed the mesenchymal epithelial transition (MET), resulting in subpopulations of CTCs that present mesenchymal and epithelial markers [35].

CTCs with mesenchymal phenotypes could have a greater propensity for tumor escape due to larger plasticity, thus facilitating the invasion and migration process [36, 37]. Moreover, CTCs with mesenchymal markers seem to be more resistant to chemotherapeutic drugs [38].

Concerning GC, Li *et al.* [16] indicated five types of cells including exclusively epithelial (E^+^) CTCs, exclusively mesenchymal (M^+^) CTCs and intermediate CTCs (E^+^ > M^+^; E^+^ = M^+^; M^+^ > E^+^), using a filtration-based method and EpCAM, CK8, CK18, CK19, Vimentin and Twist as markers. These authors observed that approximately 11% (4/35) of patients formed a subgroup exclusively with M^+^ CTCs and 29% (10/35) of patients had subgroups M^+^ and M^+^ > E^+^, just one patient had the CTCs subgroups E^+^ > M^+^ and no patient with the CTCs subgroup that was exclusively E+. Taken together, these findings demonstrated the heterogeneity of CTCs and their predominantly mesenchymal phenotype, suggesting a limitation of the methodologies that only just epithelial markers to enumerate CTCs in GC.

Although the CellSearch™ platform (Veridex LLC, Huntingdon Valley, PA, USA) uses antibodies against the adhesion molecule (EpCAM)-coated with magnetic beads, cytokeratin (CK8, CK18 and CK19) antibodies and negative staining for the CD45 (CD45-) antibody to isolate and exclusively quantify the E+ CTCs, it remains the main method used in GC studies and is the only technique approved for the enumeration and isolation of CTCs by the Food and Drug Administration (FDA) for clinical use in the prognosis of breast, colorectal and prostate cancer [39].

Additional characterization of CTCs can identify specific morphological, phenotypic and molecular features for each cancer type over time, disease stage and therapeutic definition [40]. For instance, Iwatsuki *et al.* [14] evaluated CTCs and their HER2 status in gastrointestinal cancer patients; overexpression of HER2 is a selective biomarker for treatment with the monoclonal antibody Trastuzumab in metastatic GC. In GC patients, these authors detected at least one CTC (CTC ≥ 1) in 73.5% (25/34) of samples that were 28% (7/25) HER2 positive. However, a discordant HER2 status was found between CTC-positive cases and corresponding primary tumors (HER2-positive CTCs/ HER2-negative primary tumor tissue), suggesting that primary HER2-negative tumors acquired *HER2* gene amplification in their CTCs during cancer progression. Therefore, the HER2 status of CTCs might be required as a liquid biopsy to provide personalized treatment strategies in GC.

Several studies have observed the aneuploidy of chromosome 8 in CTCs from GC patients [41, 17, 25], a frequent genetic abnormality reported in GC tumors and cell lines [42–47]. Interestingly, Li *et al.* [41] established an integrated subtraction enrichment (SET) and immunostaining-fluorescence *in situ* hybridization (iFISH) platform to detect and characterize CTCs that correlated with different ploidies of chromosome 8 in advanced GC (AGC) patients. These authors suggested that SET iFISH is significantly more sensitive than the CellSearch™ method to enumerate CTCs.

Recently, two studies performed SET-iFISH to enumerate CTCs with chromosome 8 aneuploidy before and after treatment in advanced gastric cancer (AGC) patients. Ma *et al.* [17] observed a marked expressive reduction in CTCs number with chromosome 8 amplification in patients after neoadjuvant therapy with Docetaxel/Oxaliplatin/5-FU (DOF) plus Bevacizumab compared to patients treated with DOF alone, suggesting that the addition of bevacizumab, a VEGF inhibitor, could decrease CTC counts. In addition, Li *et al.* [23] quantified CTCs and analyzed their chromosome 8 multiploidy in patients before and after therapy with the first-line (paclitaxel or cisplatin) or targeted therapy (anti-HER2 and cisplatin) and correlated these findings with the patient’s clinical prognosis. AGC patients who have an unfavorable CTC value (≥ 4 CTCs) and an unfavorable CTC multiploidy value (≥ 2 per 7.5 mL) following therapy showed a significant association with poor progression-free survival (PFS) and overall survival (OS). Moreover, patients with ≥ 10% increase in multiploid CTCs after the first 2 cycles of therapy had a greater risk of progression and mortality than patients with a decrease number of multiploidy CTCs. These studies suggested that the use of SET-iFISH to enumerate CTCs with chromosome 8 aneuploidy is an efficient method to monitor the treatment response of GC patients.

In addition, many studies have reported that the presence of CTCs in circulating tumor microemboli (CTM) confer a survival advantage in the circulatory system compared to single CTCs, which indicate poor prognosis and influence disease progression [48, 49]. In GC, Zheng *et al.* [28] observed CTCs in 59% (51/86) of GC patients in clinical stage I to IV, but CTMs were only found in 24% (10/41) of GC patients in stage IV. They concluded that the group that was CTM-positive had worse PFS and OS than the CTM-negative group (*p* < 0.001). Thus, CTM could be useful to predict prognosis in GC.

## CIRCULATING TUMOR DNA (ctDNA)

DNA fragments available in the blood stream, known as ctDNA, derived from primary tumor cells, CTCs and/or distant metastasis can reflect specific genetic cancer alterations, including mutations, amplifications, copy number variation (CNV), rearrangement and methylation [50, 51].

Accumulating evidence has demonstrated that ctDNA detection is a minimally invasive method with potential clinical applications in cancer, including i) early detection of cancer; ii) monitoring of intratumoral heterogeneity and metastasis; iii) therapeutic target identification; iv) real-time evaluation of treatment response and tumor relapse; and v) real-time evaluation of drugs resistance [52].

So far, limited studies on the identification and monitoring of ctDNA levels in GC patients have been performed. Hamakawa *et al.* [53] reported that 30% (3/10) of AGC patients had *TP53* mutations in their primary tumors and preoperative ctDNA, suggesting that identification of the *TP53* mutation (c.103delT; c.747G>C; c.166G>T) is a useful tool to monitor progression and residual disease during the clinical follow-up.

In 2016, Fang *et al.* [54] analyzed the mutational profile of eight genes (*ARID1A*, *TP53*, *PIK3CA*, *PTEN*, *AKT3*, *BRAF*, *AKT2* and *AKT1*) and the altered levels of ctDNA in 277 patients with primary gastric tumors. The authors found that *TP53, ARID1A* and *PI3KCA* were the most frequently mutated genes in AGC patients. Furthermore, they also found that patients with greater ctDNA levels were more likely to exhibit vascular invasion and a poor 5-year global survival rate than patients without detected ctDNA. Therefore, the highest ctDNA detectable levels were associated with peritoneal recurrence and a poor outcome in patients with AGC.

In addition, Shoda *et al.* [55] reported on potential of ctDNA for the detection of *HER2* amplification determined by real-time quantitative PCR (qRT-PCR) in AGC patients before surgery and during postoperative treatment, highlighting spatial and/or temporal tumor heterogeneities. Unfortunately, quantitative information using qRT-PCR is obtained from the cycle threshold (Ct) and these values can be affected by amplification imperfections that reduce efficiencies and limit the accuracy of this method for absolute quantification. On the other hand, the digital droplet PCR (ddPCR) method improves upon these limitations of nucleic acid quantification.

In 2017, Shoda *et al.* [56] showed the clinical utility of *HER2* ratios in GC patients during treatment progression and demonstrated the *HER2* status during real time evaluations using ddPCR method. Postoperative follow-ups revealed high plasma *HER2* ratios at the time of recurrence in 53.84% (7/13) cases, even in cases that were diagnosed as being *HER2* negative at the time of surgery. Overall, detection of the *HER2* ratio by digital droplet PCR (ddPCR) could provide a window of opportunity for novel decision-making treatment strategies based on *HER2* status at different periods in a clinical setting.

In a GC ctDNA meta-analysis, Gao *et al.* [57] demonstrated a significantly association between the ctDNA level based on gene methylation with the TNM stage, tumor depth, lymph node metastasis and distant metastasis in GC patients with high specificity (0.95, 95% CI 0.93–0.96) and relatively moderate sensitivity (0.62, 95% CI 0.59−0.65).

Recently, periodic mutation profiling of ctDNA from stage IV GC patients by Next Generation Sequencing (NGS) revealed the complex and heterogeneous molecular mechanisms for crizotinib resistance after two months of treatment, including reoccurrence of *MET* amplification, multiple secondary *MET* mutations (D1228, Y1230, V1092, G1163 and L1195), a remarkable increase in the relative copy number of the *FGFR2* gene as well as mutations in other downstream and related elements [58]. Crizotinib, a potent *MET* inhibitor, has demonstrated promising effects for the treatment of *MET*-amplified esophagogastric cancer [59, 60]. Moreover, *MET* amplification has been reported to occur in approximately 5% of GC patients and is targeted by crizotinib, which is currently undergoing a clinical trial in advanced *MET*-positive GC. However, tumors experienced progression shortly after crizotinib treatment [59]. Therefore, ctDNA profiling for treatment decision-making and prognosis in clinical practice have demonstrated great potential to elucidate mechanisms of resistance.

Overall, regular analysis by NGS is more expensive than ddPCR for ctDNA quantification method. Also, NGS practical use reveals an information reservoir unnecessarily for objective decision-making in clinical setting. Consequently, ddPCR could be well applied in clinical practice to identify relevant genetic aberrations in the ctDNA that facilitate GC management.

## CIRCULATING NON-CODING RNAS (ncRNAs)

Deregulated ncRNAs expression has largely been reported in the literature acting as oncogene or with tumor suppressor role in several cancers types, including GC [61–63]. Since ncRNAs are important mediators of intracellular activities with tissue specific characteristics, quantification of deregulated ncRNAs expression in blood can indicate disease state, disease progression and/or response to a particular treatment, therefore directing initial clinical practice. Secretion to the bloodstream is usually the results of cell death (necrosis or apoptosis) or due to active secretion from the cell, therefore, the expression profile reflects the primary tumor in the corresponding tissue [64].

A large number of studies have highlighted the potential importance of circulating ncRNAs as diagnostic, prognostic, and/or predictive biomarkers in cancer, mainly microRNAs (miRNAs) and long non-coding RNAs (lncRNAs). These molecules are remarkably stable as they are often incorporated into exosomes and microvesicles, thus providing resistance to RNase activity, extreme pH and multiple freeze-thaw cycles [62–63, 65–67].

### microRNAs

miRNAs are a class of single-stranded small ncRNAs of 19–25 nucleotides (nt) in length that play an essential role in the negative post-transcriptional gene regulation of at least 50% of all protein-coding gene [64]. Supplementary Table 1 summarizes a large number of circulating miRNAs as potential diagnosis and prognosis biomarkers in GC and their clinical implications [68–88].

Recently, Tsai *et al.* [80] demonstrated that miR-196a/b expression in the serum of GC patients could be more sensitive and specific for GC diagnosis than CA 19-9 (carcinoembryonic antigen 19-9) or CEA (carbohydrate antigen). Moreover, circulating miR-196a/b was also associated with TNM stage, a poor survival rate and cancer outcome, suggesting that miR-196a/b is a potential diagnostic and prognostic biomarker in GC.

Several studies also described associations between miRNA expression, *H. pylori* and EBV infection, which are well-known causes of GC [61, 62]. For instance, Shiotani *et al.* [73] observed an association between upregulated circulating miR-21 and miR-106b and *H. pylori* infection. The authors also suggested that the upregulation of both miRNAs in the serum of patients after *H. pylori* eradication could be used for the detection of high risk GC in individuals with extensive atrophy.

A single candidate approach can reveal deregulated miRNA, however, the search for a miRNA signature profile that can predict prognosis and monitor cancer progression has been a common focus of many studies. Among these studies, it was reported that the miRNA profile in the serum/plasma of patients with GC displayed unique miRNA changes or a miRNAs signature.

Using MiSeq sequencing, Jiang *et al.* [89] performed an initial screening of serum miRNAs in ten GC patients with lymph node metastasis (LNM+), ten patients without lymph node metastasis (LNM-) and ten healthy controls. Then, the candidate miRNAs (miR-501-3p, miR-143-3p, miR-451, and miR-146a) were validated in serum samples from 73 controls, 103 LNM+ and 103 LNM- patients by qRT-PCR. Prediction of LNM+ in GC restricted to the mucosa prior to surgery with a circulating miRNA panel could help determine the need for surgical lymph node resection. This would allow endoscopic mucosal resection, a less invasive treatment, to be immediately conducted without delay to provide more effective treatment for early gastric tumors. On the other hand, endoscopic resection of tumors should be avoided when the miRNA panel to indicates LNM+. In the case, surgical resection with an extensive lymphadenectomy would be recommended for a better outcome in GC patients.

Despite numerous efforts, no consensus has been found for miRNA biomarkers that can be incorporated into GC clinical practice. For this occur, a number of obstacles must be overcome, for example, the quantification of miRNAs can suffer from variations due to inadequate processing, storage, RNA extraction, and reference genes choice for qRT-PCR quantification. Variation is such a problem that and even differences between serum and plasma miRNA quantification have been observed [86, 69, 90–93]. Moreover, there is no unique protocol to control for these parameters. Divergences in the analysis of the circulating miRNA make it difficult for perform a comparison among them.

### Long non-coding RNAs

lncRNAs comprise a diverse class of RNA transcripts >200 nt in length. They regulate gene expression through a variety of transcriptional and post-transcriptional mechanisms, including i) chromatin modification and remodeling; ii) direct transcriptional regulation; iii) regulation of RNA processing events such as splicing, editing, localization, translation and turnover/degradation;, iv) induction of DNA methyltransferases; v) protein scaffolding; vi) modulation of miRNA regulation; vii) miRNA precursor processing; viii) regulation of translation; and ix) protein binding [94].

Similar to miRNAs, a number of circulating lncRNAs also have emerged as diagnostic and/or prognostic biomarkers in GC (Supplementary Table 2) [95–103]. For instance, HULC (highly up-regulated in liver cancer) was significantly higher in the serum of GC patients than healthy controls. Interestingly, the serum HULC level was significantly decreased in post-treatment patients to a level similar to that of healthy individuals. In addition, serum HULC levels expression was associated with tumor size, lymph node metastasis, distant metastasis and *H. pylori*, a strong risk factor for both GC development and progression. Furthermore, a ROC curve to evaluate the diagnostic utility of HULC revealed that serum HULC levels provides a more powerful differential ability than CEA and CA72-4, follow-up detection and Kaplan-Meier curve analysis showed that HULC is a good predictor of GC prognosis. Taken together, these findings indicate that HULC may be a potential tumor biomarker for early diagnosis, progression monitoring and GC prognosis of GC [100].

According to Chao *et al.* [104], elevated circulating levels of AA174084 were associated with invasion and lymph node metastasis in GC patients. Their levels dropped markedly on day 15 after surgery compared to preoperative levels. However, the measurement of plasma-based AA174084 has obvious limitations, because AA174084 levels in plasma do not differ between healthy individuals and GC patients. Thus, the authors suggested that AA174084 may have potential as a prognostic biomarker for GC.

Several studies investigated the levels of circulating H19 (H19, imprinted maternally expressed transcript) from GC patients as potential diagnostic biomarker [96–98]. Arita *et al.* [96] found that H19 levels were significantly higher in the plasma of GC patients than in healthy controls. However, there was no correlation between plasma H19 levels and the clinicopathological factors of these GC patients. In comparison with the levels in pre- and postoperative paired plasma samples, H19 levels were significantly lower in postoperative plasma.

In 2015, Zhou *et al.* [97] validated the expression of eight lncRNAs (HOTAIR, CCAT1, PVT1, H19, MALAT1, MRUL, GHET1 and HULC) by test-scale analyses in tissue and plasma using qRT-PCR. Among them, H19 and another five lncRNAs (HOTAIR, PVT1, MALAT1, GHET1 and HULC) were significantly higher in tumor tissues compared to matched normal samples. Of these lncRNAs, only H19, MALAT1 and HOTAIR were significantly higher in the plasma of ten GC patients compared to ten healthy controls. Among these three lncRNAs, only H19 expression was significantly higher in GC patient plasma compared to heathy controls, when plasma lncRNAs levels were examined on a large scale using plasma from 70 GC patients and 70 healthy controls. That analysis involved the comparison of plasma H19 concentrations in paired plasma obtained from pre- and postoperative samples; H19 levels were significantly reduced postoperatively in patients with high preoperative plasma H19. Clearly, these findings demonstrated that plasma levels of H19 are useful as a potential biomarker for the diagnosis of GC, particularly for early tumor screening.

Many of the obstacles that exist for the effective application of circulating lncRNAs in GC clinical practice are similar to those described for circulating miRNAs.

## FUTURE DIRECTIONS

**Figure 1:**
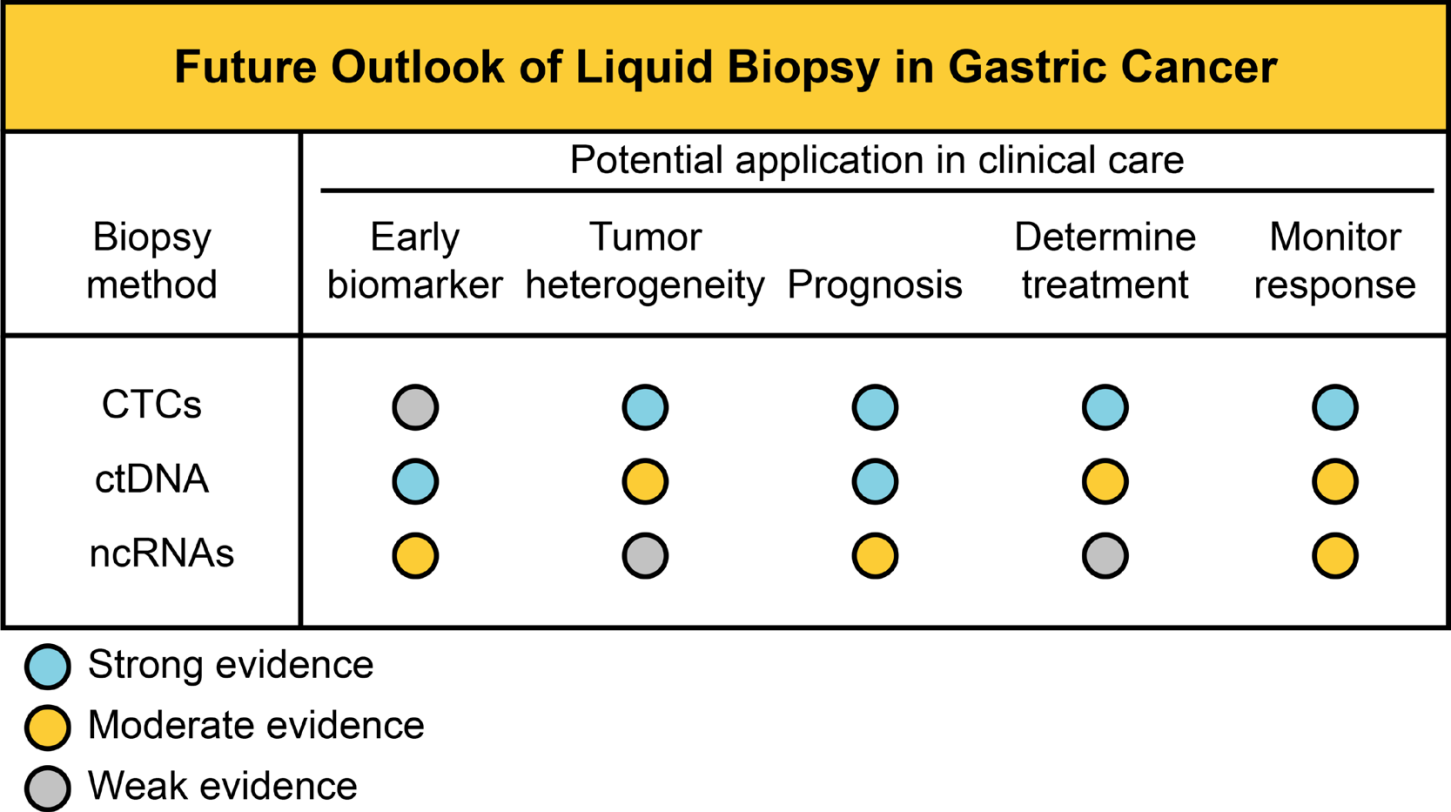
Potential application of liquid biopsies in GC management Establishment of standard analysis of CTCs, ctDNA and circulating ncRNAs in the future.

Liquid biopsy approaches have enormous implications for cancer, ranging from early diagnosis to the monitoring of treatment response, and have transformed clinical care. Currently, the liquid biopsy does not replace the conventional biopsy, however, it has been applied to tumor growth control and in deciding on therapeutic choice to improve the overall survival rate of patient with different cancer types. Nevertheless, liquid biopsies remain removed from GC clinical management. Figure 1 summarizes the putative outlook of liquid biopsy use and their potential application in clinical care in GC management (classified as strong, moderate and weak evidence).

**Figure 2:**
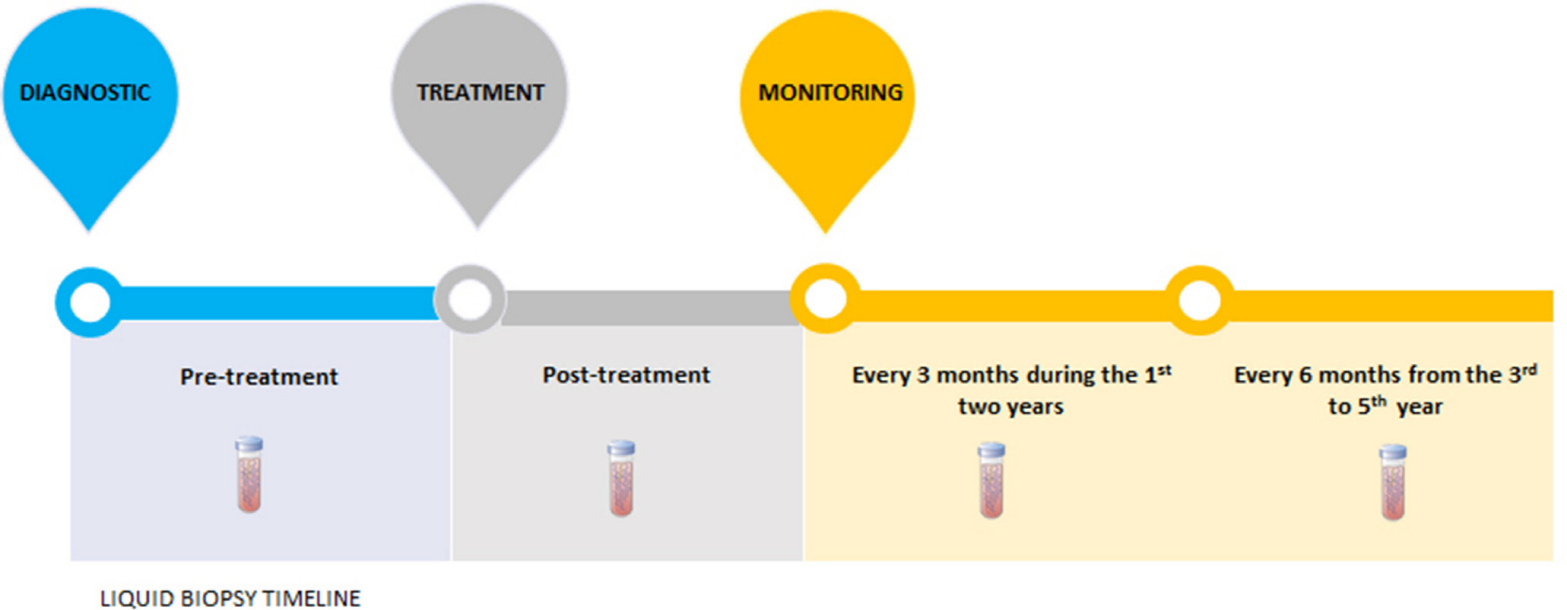
Liquid biopsies timeline At the diagnostic, the pretreatment ctDNA demonstrates the molecular characteristics of the tumor, as well as the patient's prognosis. After treatment, ctDNA quantification could measure the treatment's efficacy, since ctDNA bloodstream half-time is about 1-2.4hours. In case of advanced gastric tumors, CTCs analysis could also be used in the same manner. Moreover, monitoring of ctDNA or CTCs, every three months during the first two years and every six months from the third to fifth year, could evaluate therapeutic response and recurrence disease before the patients shows clinical symptoms or metastasis is observed by computed tomography.

In GC, we believe that the earliest use of liquid biopsy in clinical practice should focus on therapies that target detection and monitoring. Indeed, *HER2* status in CTCs or ctDNA has emerged as a therapeutic marker of effective molecular targeted therapy and therapeutic response monitoring in GC patients [14, 56, 57]. Figure 2 shows timeline of CTC and ctDNA analysis in pre-treatment and post-treatment (immediately and monitoring) period of GC patients.

Before liquid biopsies are incorporated into clinical practice as a precision medicine tool to drive GC management, pre-analytical steps must be standardized in order to ensure reproducible processing techniques. Moreover, analytical steps must be validated, such as the enumeration of CTCs and ctDNA, the quantification of circulating ncRNAs, subsequent CTCs characterization and genetic or epigenetic alterations in ctDNA analysis. Finally, CTCs markers or assays applied to circulating ncRNAs or ctDNA measurements must have strong and reproducible sensitivity and specificity, beyond having the suitable internal and external quality controls.

Consequently, many questions remain about liquid biopsies from blood that require resolution: i) how should the blood samples be collected to ensure quality biomarker detection; ii) which is the ideal method and marker for CTC enumeration and characterization; iii) what gene alterations are key for ctDNA measurement; iv) what reference genes are stable and suitable for circulating ncRNA measurement in GC patients; v) what criteria should be adopted for the CTC, ctDNA and circulating ncRNA validation analysis.

## SUPPLEMENTARY MATERIALS TABLES






