# Supplementary material for: Liquid biopsy provides new insights into gastric cancer
Source: Oncotarget. 2018 Feb 21;9(19):15144–56. doi: 10.18632/oncotarget.24540 (PMC5871105; doi:10.18632/oncotarget.24540)
Supplement: Supplementary file 1 [file oncotarget-09-15144-s001.pdf]

Liquid biopsy provides new insights into gastric cancer

SUPPLEMENTARY MATERIALS

Supplementary Table 1: Deregulated miRNAs and their clinical implication in GC. See Supplementary_Table_1

Supplementary Table 2: Circulating lncRNAs as potential biomarkers and their clinical implications in GC. See Supplementary_Table_2

SUPPLEMENTARY REFERENCES

1. Huang Z, Zhu D, Wu L, He M, Zhou X, Zhang L, Zhang H, Wang W, Zhu J, Cheng W, Chen Y, Fan Y, Qi L, et al. Six Serum-Based miRNAs as Potential Diagnostic Biomarkers for Gastric Cancer. Cancer Epidemiol Biomarkers Prev. 2017; 26:188–96. https://doi.org/10.1158/1055-9965.EPI-16-0607.
2. Wang H, Wang L, Wu Z, Sun R, Jin H, Ma J, Liu L, Ling R, Yi J, Wang L, Bian J, Chen J, Li N, et al. Three dysregulated microRNAs in serum as novel biomarkers for gastric cancer screening. Med Oncol. 2014; 31:298. https://doi.org/10.1007/s12032-014-0298-8.
3. Zhou H, Guo JM, Lou YR, Zhang XJ, Zhong FD, Jiang Z, Cheng J, Xiao BX. Detection of circulating tumor cells in peripheral blood from patients with gastric cancer using microRNA as a marker. J Mol Med (Berl). 2010; 88:709–17. https://doi.org/10.1007/s00109-010-0617-2.
4. Yang R, Fu Y, Zeng Y, Xiang M, Yin Y, Li L, Xu H, Zhong J, Zeng X. Serum miR-20a is a promising biomarker for gastric cancer. Biomed Rep. 2017; 6:429–34. https://doi.org/10.3892/br.2017.862.
5. Shiotani A, Murao T, Kimura Y, Matsumoto H, Kamada T, Kusunoki H, Inoue K, Uedo N, Iishi H, Haruma K. Identification of serum miRNAs as novel non-invasive biomarkers for detection of high risk for early gastric cancer. Br J Cancer. 2013; 109:2323–30. https://doi.org/10.1038/bjc.2013.596.
6. Zhang R, Wang W, Li F, Zhang H, Liu J. MicroRNA-106b~25 expressions in tumor tissues and plasma of patients with gastric cancers. Med Oncol. 2014; 31:243. https://doi.org/10.1007/s12032-014-0243-x.
7. Qiu X, Zhang J, Shi W, Liu S, Kang M, Chu H, Wu D, Tong N, Gong W, Tao G, Zhao Q, Qiang F, Zhu H, et al. Circulating MicroRNA-26a in Plasma and Its Potential Diagnostic Value in Gastric Cancer. PLoS One. 2016; 11:e0151345. https://doi.org/10.1371/journal.pone.0151345.
8. Liu R, Zhang C, Hu Z, Li G, Wang C, Yang C, Huang D, Chen X, Zhang H, Zhuang R, Deng T, Liu H, Yin J, et al. A five-microRNA signature identified from genome-wide serum microRNA expression profiling serves as a fingerprint for gastric cancer diagnosis. Eur J Cancer. 2011; 47:784–91. https://doi.org/10.1016/j.ejca.2010.10.025.
9. Chen Q, Ge X, Zhang Y, Xia H, Yuan D, Tang Q, Chen L, Pang X, Leng W, Bi F. Plasma miR-122 and miR-192 as potential novel biomarkers for the early detection of distant metastasis of gastric cancer. Oncol Rep. 2014; 31:1863–70. https://doi.org/10.3892/or.2014.3004.
10. Kim SY, Jeon TY, Choi CI, Kim DH, Kim DH, Kim GH, Ryu DY, Lee BE, Kim HH. Validation of circulating miRNA biomarkers for predicting lymph node metastasis in gastric cancer. J Mol Diagn. 2013; 15:661–69. https://doi.org/10.1016/j.jmoldx.2013.04.004.
11. Gorur A, Balci Fidanci S, Dogruer Unal N, Ayaz L, Akbayir S, Yildirim Yaroglu H, Dirlik M, Serin MS, Tamer L. Determination of plasma microRNA for early detection of gastric cancer. Mol Biol Rep. 2013; 40:2091–96. https://doi.org/10.1007/s11033-012-2267-7.
12. Tsai MM, Wang CS, Tsai CY, Huang CG, Lee KF, Huang HW, Lin YH, Chi HC, Kuo LM, Lu PH, Lin KH. Circulating microRNA-196a/b are novel biomarkers associated with metastatic gastric cancer. Eur J Cancer. 2016; 64:137–48. https://doi.org/10.1016/j.ejca.2016.05.007.
13. Li C, Li JF, Cai Q, Qiu QQ, Yan M, Liu BY, Zhu ZG. MiRNA-199a-3p: A potential circulating diagnostic biomarker for early gastric cancer. J Surg Oncol. 2013; 108:89–92. https://doi.org/10.1002/jso.23358.
14. Valladares-Ayerbes M, Reboredo M, Medina-Villaamil V, Iglesias-Díaz P, Lorenzo-Patiño MJ, Haz M, Santamarina I, Blanco M, Fernández-Tajes J, Quindós M, Carral A, Figueroa A, Antón-Aparicio LM, Calvo L. Circulating miR-200c as a diagnostic and prognostic biomarker for gastric cancer. J Transl Med. 2012; 10:186. https://doi.org/10.1186/1479-5876-10-186.
15. Imaoka H, Toiyama Y, Okigami M, Yasuda H, Saigusa S, Ohi M, Tanaka K, Inoue Y, Mohri Y, Kusunoki M. Circulating microRNA-203 predicts metastases, early recurrence, and poor prognosis in human gastric cancer. Gastric Cancer. 2016; 19:744–53. https://doi.org/10.1007/s10120-015-0521-0.
16. Zhang WH, Gui JH, Wang CZ, Chang Q, Xu SP, Cai CH, Li YN, Tian YP, Yan L, Wu B. The identification of miR-375 as a potential biomarker in distal gastric adenocarcinoma. Oncol Res. 2012; 20:139–47. https://doi.org/10.3727/096504012X13522227232156.
17. Song MY, Pan KF, Su HJ, Zhang L, Ma JL, Li JY, Yuasa Y, Kang D, Kim YS, You WC. Identification of serum microRNAs as novel non-invasive biomarkers for early detection of gastric cancer. PLoS One. 2012; 7:e33608. https://doi.org/10.1371/journal.pone.0033608.
18. Wu J, Li G, Yao Y, Wang Z, Sun W, Wang J. MicroRNA-421 is a new potential diagnosis biomarker with higher sensitivity and specificity than carcinoembryonic antigen and cancer antigen 125 in gastric cancer. Biomarkers. 2015; 20:58–63. https://doi.org/10.3109/1354750X.2014.992812.
19. Shin VY, Ng EK, Chan VW, Kwong A, Chu KM. A three-miRNA signature as promising non-invasive diagnostic marker for gastric cancer. Mol Cancer. 2015; 14:202. https://doi.org/10.1186/s12943-015-0473-3.
20. Liu X, Kwong A, Sihoe A, Chu KM. Plasma miR-940 may serve as a novel biomarker for gastric cancer. Tumour Biol. 2016; 37:3589–97. https://doi.org/10.1007/s13277-015-4019-5.
21. Shao Y, Ye M, Jiang X, Sun W, Ding X, Liu Z, Ye G, Zhang X, Xiao B, Guo J. Gastric juice long noncoding RNA used as a tumor marker for screening gastric cancer. Cancer. 2014; 120:3320–28. https://doi.org/10.1002/cncr.28882.
22. Arita T, Ichikawa D, Konishi H, Komatsu S, Shiozaki A, Shoda K, Kawaguchi T, Hirajima S, Nagata H, Kubota T, Fujiwara H, Okamoto K, Otsuji E. Circulating long non-coding RNAs in plasma of patients with gastric cancer. Anticancer Res. 2013; 33:3185–93.
23. Zhou X, Yin C, Dang Y, Ye F, Zhang G. Identification of the long non-coding RNA H19 in plasma as a novel biomarker

for diagnosis of gastric cancer. Sci Rep. 2015; 5:11516. https://doi.org/10.1038/srep11516.

24. Yang T, Zeng H, Chen W, Zheng R, Zhang Y, Li Z, Qi J, Wang M, Chen T, Lou J, Lu L, Zhou T, Dai S, et al. Helicobacter pylori infection, H19 and LINC00152 expression in serum and risk of gastric cancer in a Chinese population. Cancer Epidemiol. 2016; 44:147–53. https://doi.org/10.1016/j.canep.2016.08.015.

25. Li Q, Shao Y, Zhang X, Zheng T, Miao M, Qin L, Wang B, Ye G, Xiao B, Guo J. Plasma long noncoding RNA protected by exosomes as a potential stable biomarker for gastric cancer. Tumour Biol. 2015; 36:2007–12. https://doi.org/10.1007/s13277-014-2807-y.

26. Jin C, Shi W, Wang F, Shen X, Qi J, Cong H, Yuan J, Shi L, Zhu B, Luo X, Zhang Y, Ju S. Long non-coding RNA HULC as a novel serum biomarker for diagnosis and prognosis prediction of gastric cancer. Oncotarget. 2016; 7:51763–72. https://doi.org/10.18632/oncotarget.10107.

27. Dong L, Qi P, Xu MD, Ni SJ, Huang D, Xu QH, Weng WW, Tan C, Sheng WQ, Zhou XY, Du X. Circulating CUDR, LSINCT-5 and PTENP1 long noncoding RNAs in sera distinguish patients with gastric cancer from healthy controls. Int J Cancer. 2015; 137:1128–35. https://doi.org/10.1002/ijc.29484.

28. Liu Z, Shao Y, Tan L, Shi H, Chen S, Guo J. Clinical significance of the low expression of FER1L4 in gastric cancer patients. Tumour Biol. 2014; 35:9613–17. https://doi.org/10.1007/s13277-014-2259-4.

29. Zeng X, Shi H, Wang J, Cui S, Tang H, Zhang X. Long noncoding RNA aberrant expression profiles after cytoreductive surgery and hyperthermic intraperitoneal chemotherapy of AGC ascertained by microarray analysis. Tumour Biol. 2015; 36:5021–29. https://doi.org/10.1007/s13277-015-3153-4.
