# Supplementary material for: Liquid biopsy provides new insights into gastric cancer
Source: Oncotarget. 2018 Feb 21;9(19):15144–56. doi: 10.18632/oncotarget.24540 (PMC5871105; doi:10.18632/oncotarget.24540)
Supplement: Supplementary file 2 [file oncotarget-09-15144-s002.docx]

**Supplementary Table 1: Deregulated miRNAs and their clinical implication in GC**

| **Circulating**  **miRNAs** | **Expression** | **Samples** | **Potential Biomarker** | **Method** | **Clinical implication** | **Reference** |
| --- | --- | --- | --- | --- | --- | --- |
| miR-10b-5p | ↑ | 154 GC  120 C  Serum | Diagnostic | microarray  qRT-PCR | OS | [1] |
| miR-16 | ↑ | 50 GC  47 C  Plasma | Diagnostic  Prognostic | qRT-PCR | TNM Stage  Metastasis | [2] |
| miR-17 | ↑ | 90 GC  27 C  Serum | Diagnostic  Prognostic | qRT-PCR | CTCs  Detection | [3] |
| miR-20a-3p | ↑ | 154 GC  120C  Serum | Diagnostic | microarray  qRT-PCR | - | [1] |
| miR-20a-5p | ↑ | 12 GC  12 C  Serum | Diagnostic  Prognostic | microarray  qRT-PCR | Age  TNM Stage  Degree of differentiation  LNM | [4] |
| miR-21 | ↑ | 87 GCNC  114 C  Serum | Diagnostic  Prognostic | qRT-PCR | *H. pylori* Infection  Risk for Early GC | [5] |
| miR-25 | ↑ | 40 PreO GC  20 PostO GC  Plasma | Diagnostic  Prognostic | qRT-PCR | TNM Stage  Invasion | [6] |
| miR-26a | ↓ | 285 GC  285 C  Plasma | Diagnostic | microarray  qRT-PCR | - | [7] |
| miR- 27a | ↑ | 164 GC  127 C  Serum | Diagnostic | Solexa sequencing | - | [8] |
| miR-93 | ↑ | 40 PreO GC  20 PostO GC  Plasma | Diagnostic  Prognostic | qRT-PCR | TNM Stage  Invasion | [6] |
| miR-106a | ↑ | 90 GC  27 C  Serum | Diagnostic Prognostic | qRT-PCR | CTCs  Detection | [3] |
| miR-106b | ↑ | 87 GCNC  114C  Serum | Diagnostic  Prognostic | qRT-PCR | *H. pylori* Infection  Risk for Early GC | [5] |
|  | ↑ | 40 PreO GC 20 PostO GC  Plasma | Diagnostic  Prognostic | qRT-PCR | TNM Stage  Invasion | [6] |
| miR-122 | ↓ | 36 GCDM  36 GCNDM  36 C  Plasma | Prognostic | qRT-PCR | Metastasis | [9] |
|  | ↑ | 154 GC  120 C  Serum | Diagnostic | microarray  qRT-PCR | - | [1] |
| miR-142-3p | ↓ | 285 GC  285 C  Plasma | Diagnostic | microarray  qRT-PCR | - | [7] |
| miR-148a | ↓ | 285 GC  285 C  Plasma | Diagnostic | microarray  qRT-PCR | - | [7] |
|  | ↑ | 16 LNM +  15 LNM-  15 C  Serum | Prognostic | qRT-PCR | LNM | [10] |
| miR-185-5p | ↑ | 154 GC  120 C  Serum | Diagnostic | Microarray  qRT-PCR | - | [1] |
| miR-192 | ↑ | 36 GCDM  36 GCNDM  36 C  Pma | Prognostic | qRT-PCR | Metastasis | [9] |
| miR-195 | ↓ | 285 GC  285 C  Plasma | Diagnostic | Microarray  qRT-PCR | - | [7] |
| miR-195-5p | ↑ | 154 GC  120 C  Serum | Diagnostic | Microarray  qRT-PCR | - | [1] |
|  | ↓ | 20 GC  190 C  Plasma | Diagnostic | qRT-PCR | - | [11] |
| miR-196a/b | ↑ | 98 GC  126 C  Plasma | Diagnostic  Prognostic | qRT-PCR | TNM Stage  Metastasis  SO | [12] |
| miR-199a-3p | ↑ | 30 GC  70 C  Plasma | Diagnostic | qRT-PCR | - | [13] |
| miR-200c | ↑ | 52 GC  15 C  Whole blood | Diagnostic Prognostic | qRT-PCR | LNM  SO | [14] |
| miR-203 | ↓ | 130 GC 22C  Serum | Prognostic | qRT-PCR | TNM Stage  LNM  Peritoneal Metastasis | [15] |
| miR-223 | ↑ | 50 GC  47 C  Plasma | Diagnostic  Prognostic | qRT-PCR | TNM Stage  Metastasis | [2] |
| miR-296-5p | ↑ | 154 GC  120C  Serum | Diagnostic | Microarray  qRT-PCR | OS | [1] |
| miR-375 | ↓ | 20 DGAC  20 C  Serum | Diagnostic | qRT-PCR | DGAC  Detection | [16] |
| miR-376c | ↑ | 58 GC  Serum | Diagnostic | qRT-PCR | - | [17] |
| miR-421 | ↑ | 90 GC  90 C  Serum  PBMCs | Diagnostic | qRT-PCR | - | [18] |
| miR-627 | ↑ | 58 CG  46C  Plasma | Diagnostic | Microarray  qRT-PCR | - | [19] |
| miR-629 | ↑ | 58 CG  46C  Plasma | Diagnostic | Microarray  qRT-PCR | - | [19] |
| miR-652 | ↑ | 58 CG  46C  Plasma | Diagnostic | Microarray  qRT-PCR | - | [19] |
| miR-744 | ↑ | 58 GC  Serum | Diagnostic | qRT-PCR | - | [17] |
| miR-940 | ↓↑ | 115 GC  105C  Plasma | Diagnostic  Prognostic | Microarray  qRT-PCR | Metastasis | [20] |

GC: gastric cancer group; C: control group; DGAC: diffuse gastric adenocarcinoma; GCNC: gastric cancer non-cardiac type; EBV: Epstein Barr virus; LNM: lymph nodes metastasis; CTC: circulating tumor cell; GCDM: gastric cancer with distant metastasis; GCNDM: gastric cancer without distant metastasis; OS: overall survival; PBMNCs: peripheral blood mononuclear cells; PreO GC: preoperative gastric cancer; PostO GC: postoperative gastric cancer; SO: survival outcome.
