# Supplementary material for: Liquid biopsy provides new insights into gastric cancer
Source: Oncotarget. 2018 Feb 21;9(19):15144–56. doi: 10.18632/oncotarget.24540 (PMC5871105; doi:10.18632/oncotarget.24540)
Supplement: Supplementary file 3 [file oncotarget-09-15144-s003.docx]

**Supplementary Table 2: Circulating lncRNAs as potential biomarkers and their clinical implications in GC**

| **lncRNA** | **Expression** | **Samples** | **Potential Biomarker** | **Clinical Implication** | **Reference** |
| --- | --- | --- | --- | --- | --- |
| AA174084 | ↓ | 83 PreO GC  103 PostO GC  29 Dys  120 C  Plasma | Prognostic | Invasion  LNM | [21] |
| H19 | ↑ | 43GC  33 C  Plasma | Diagnostic | - | [22] |
|  | ↑ | 70 GC  70 C  Plasma | Diagnostic | - | [23] |
|  | ↑ | 133 GC  152 C  Serum | Diagnostic | *H. pylori* infection | [24] |
| LINC00152 | ↑ | 79 GC  31 Dys  81 C  Plasma | Diagnostic | - | [25] |
|  | ↑ | 133 GC  152 C  Serum | Diagnostic | *H. pylori* infection | [24] |
| HULC | ↑ | 173 GC  30 GP  30 IM or HAH  110 C  Serum | Diagnostic  Prognostic | Tumor size  LNM  Distant metastasis  Staging  *H. pylori* infection | [26] |
| PTENP1 | ↓ | 73 GC  15 GU  86 C  Serum | Diagnostic | _ | [27] |
| LSINCT-5 | ↓ | 73 GC  15 GU  86 C  Serum | Diagnostic | _ | [27] |
| CUDR | ↓ | 73 GC  15 GU  86 C  Serum | Diagnostic | _ | [27] |
| FER1L4 | ↓ | 83 GC  80 C  Plasma | Prognostic | Tumor size  Differentiation  LNM  Distant metastasis  Venous invasion  Perineural invasion | [28] |
| BC031243 | ↑ | 6 AGC  (CRS+HIPEC) Serum | Diagnostic | _ | [29] |
| RP11-356I2.2 | ↑ | 6 AGC  (CRS+HIPEC) Serum | Diagnostic | _ | [29] |

AG: atrophic gastritis patients; AGC: advanced gastric cancer patients; C: control group; CPEC: hyperthermic intraperitoneal chemotherapy group; CRS: cytoreductive surgery group; Dys: dysplasia patients; GC: gastric cancer patients; GP: gastric polyps patients; GU: gastric ulcers patients; HAH: high atypical hyperplasia patients; IM: intestinal metaplasia patients; LNM: lymph node metastasis; NMMG: normal mucosa or minimal gastritis; PostO GC: postoperative gastric cancer; PreO GC: preoperative gastric cancer.
